# The majority of SARS-CoV-2-specific antibodies in COVID-19 patients with obesity are autoimmune and not neutralizing

**DOI:** 10.1038/s41366-021-01016-9

**Published:** 2021-11-07

**Authors:** Daniela Frasca, Lisa Reidy, Maria Romero, Alain Diaz, Carolyn Cray, Kristin Kahl, Bonnie B. Blomberg

**Affiliations:** 1 Department of Microbiology and Immunology, University of Miami Miller School of Medicine, Miami, FL USA; 2 Sylvester Comprehensive Cancer Center, University of Miami Miller School of Medicine, Miami, FL USA; 3 Department of Pathology & Laboratory Medicine, University of Miami Miller School of Medicine, Miami, FL USA

**Keywords:** Immunology, Endocrinology

## Abstract

**Background/objectives:**

Obesity decreases the secretion of SARS-CoV-2-specific IgG antibodies in the blood of COVID-19 patients. How obesity impacts the quality of the antibodies secreted, however, is not understood. Therefore, the objective of this study is to evaluate the presence of neutralizing versus autoimmune antibodies in COVID-19 patients with obesity.

**Subjects/methods:**

Thirty serum samples from individuals who tested positive for SARS-CoV-2 infection by RT-PCR were collected from inpatient and outpatient settings. Of these, 15 were lean (BMI < 25) and 15 were obese (BMI ≥ 30). Control serum samples were from 30 uninfected individuals, age-, gender-, and BMI-matched, recruited before the current pandemic. Neutralizing and autoimmune antibodies were measured by ELISA. IgG autoimmune antibodies were specific for malondialdehyde (MDA), a marker of oxidative stress and lipid peroxidation, and for adipocyte-derived protein antigens (AD), markers of virus-induced cell death in the obese adipose tissue.

**Results:**

SARS-CoV-2 infection induces neutralizing antibodies in all lean but only in few obese COVID-19 patients. SARS-CoV-2 infection also induces anti-MDA and anti-AD autoimmune antibodies more in lean than in obese patients as compared to uninfected controls. Serum levels of these autoimmune antibodies, however, are always higher in obese versus lean COVID-19 patients. Moreover, because the autoimmune antibodies found in serum samples of COVID-19 patients have been correlated with serum levels of C-reactive protein (CRP), a general marker of inflammation, we also evaluated the association of anti-MDA and anti-AD antibodies with serum CRP and found a positive association between CRP and autoimmune antibodies.

**Conclusions:**

Our results highlight the importance of evaluating the quality of the antibody response in COVID-19 patients with obesity, particularly the presence of autoimmune antibodies, and identify biomarkers of self-tolerance breakdown. This is crucial to protect this vulnerable population at higher risk of responding poorly to infection with SARS-CoV-2 than lean controls.

## Introduction

The novel single-stranded RNA coronavirus SARS-CoV-2 (severe acute respiratory syndrome corona virus-2) emerged in the last months of 2019, caused the worldwide coronavirus disease 2019 (COVID-19) pandemic, and was responsible for different clinical manifestations ranging from mild disease to severe respiratory tract infections, multiorgan failure, and death. The severe manifestations of the disease are associated with an exuberant inflammatory response and the development of a condition known as cytokine storm [1]. Published data have indicated that inflammaging, the chronic low-grade systemic inflammation [2], is a major cause of the cellular and molecular changes induced by SARS-CoV-2 and can be responsible for the highest number of deaths [3]. In addition, inflammaging induces chronic immune activation (IA) and dysfunctional immunity [4].

Resolution of SARS-CoV-2 infection requires both innate and adaptive immune responses that lead to the clearance and elimination of the virus from the organism. B cells contribute to this process by producing neutralizing antibodies that prevent the spread of infectious virions, control virus dissemination, and reduce tissue damage. Neutralizing antibodies generated against the Spike glycoprotein of the SARS-CoV-1 in the 2002–2003 pandemic have shown efficacy in protecting from severe disease [5]. Moreover, during the current pandemic, it has been shown that neutralizing antibodies against the Spike glycoprotein of SARS-CoV-2, found in plasma from convalescent COVID-19 patients, induced fast recovery when transfused into critically ill patients [6–8].

Obesity is an inflammatory condition associated with inflammaging and chronic IA, contributing to functional impairment of immune cells, and decreased immunity. Obese individuals have been shown to respond poorly to infections [9–11], vaccination [12–14], and therapies for autoimmune conditions [15]. Therefore, obesity represents an additional risk factor for COVID-19 patients. A strong association between obesity, obesity-associated comorbidities, and severe outcomes of COVID-19 has indeed been shown [16], with adult COVID-19 symptomatic patients with Body Mass Index (BMI) >30 showing higher admission to acute and critical care compared to lean and overweight individuals (BMI < 30) [17]. These results have been confirmed in part in a multi-site prospective cohort of non-hospitalized individuals in which obesity was found to be associated with the presence of multiple COVID-19 symptoms [18] However, in this cohort obesity was not associated with increased risk of infection.

We have previously evaluated the effects of obesity on the secretion of SARS-CoV-2-specific immunoglobulin G (IgG) antibodies in the blood of lean and overweight/obese COVID-19 patients, and we have shown that SARS-CoV-2 IgG antibodies are negatively associated with BMI in COVID-19 patients, as expected based on the known effects of overweight/obesity on humoral immunity [19]. In this study, we have evaluated the quality of the antibody response in lean (BMI < 25) and obese (BMI > 30) COVID-19 patients, as compared to age-, gender-, and BMI-matched uninfected controls, without previous history of autoimmunity. It has been recently shown that the sera of adult COVID-19 patients contain antibodies with autoimmune specificities [20]. We measured the presence of neutralizing antibodies and autoimmune antibodies specific for malondialdehyde (MDA), which is used as a marker of oxidative stress and lipid peroxidation, and for adipocyte-derived protein antigens (AD), established markers of cell death in the obese adipose tissue (AT). This study included uninfected controls since obesity per se is associated with the secretion of autoimmune antibodies, as previously demonstrated [21–23]. We hypothesized that SARS-CoV-2 infection in COVID-19 patients with obesity induces oxidative stress and tissue damage, leading to cell death and release of intracellular antigens that are not known to be immunogenic autoantigens, and more in obese than in lean patients.

## Materials/subjects and methods

### Participants

Experiments were performed using serum samples isolated from individuals who tested positive for SARS-CoV-2 RNA by reverse transcriptase-polymerase chain reaction (RT-PCR) of nasopharyngeal swab samples. Only serum samples were collected from patients, and samples used in this study were discarded clinical samples, only taken for routine clinical care purposes. Samples were de-identified before being used in this study. In total, 30 serum samples from individuals tested positive for SARS-CoV-2 infection by RT-PCR, 15 lean (BMI < 25) and 15 obese (BMI ≥ 30), were collected from both inpatient and outpatient settings and frozen until testing was performed. Both lean and obese patients had multiple respiratory symptoms (high fever, cough, shortness of breath, hypoxia), as evaluated at the time of hospital admission, but none was admitted to Intensive Care Unit. The research was approved and reviewed by the Institutional Review Board (IRB, protocol #20200504, PI Dr. Reidy) at the University of Miami, which reviews all human research conducted under the auspices of the University of Miami.

Control serum samples were from 30 uninfected individuals, age-, gender-, and BMI-matched, recruited at the University of Miami before the current pandemic (IRB, protocols #20070481 and #20160542, PI Dr. Frasca). Both lean and obese uninfected controls were screened for diseases known to alter the immune response or for consumption of medications that could alter the immune response. We excluded subjects with autoimmune diseases, congestive heart failure, cardiovascular disease, chronic renal failure, malignancies, renal or hepatic diseases, infectious disease, trauma or surgery, pregnancy, or documented current substance and/or alcohol abuse.

The characteristics of COVID-19 patients and controls are in Table 1. Both COVID-19 patients and uninfected controls were without prior history of autoimmunity.

**Table 1:** Characteristics of enrolled participants.

	Uninfected		SARS-CoV-2 infected	
	Lean	Obese	Lean	Obese
Age, mean±SE	53 ± 4	50 ± 2	64 ± 9^a^	55 ± 4^b^
BMI, mean±SE	22 ± 1	37 ± 2^c^	22 ± 3	35 ± 2^d^
Males	7	8	7	6
Females	8	7	8	9

^a^Mean comparison of the ages in lean uninfected versus SARS-CoV-2-infected: *p* = 0.0973.

^b^Mean comparison of the ages in obese uninfected versus SARS-CoV-2-infected: *p* = 0.8094.

^c^Mean comparison of BMI of lean and obese uninfected: *p* < 0.0001.

^d^Mean comparison of BMI of lean and obese SARS-CoV-2-infected: *p* < 0.0001.

### SARS-CoV-2 tests

SARS-CoV-2 RNA was detected by RT-PCR of nasopharyngeal swab samples. RT-PCR tests were performed at the clinical laboratories of the Department of Pathology & Laboratory Medicine using either the Diasorin or the BDMax assay and reagents. Depending on submission type (symptomatic or asymptomatic), the sample was assigned and tested per the manufacturer guidelines.

### Enzyme-linked immunosorbent assay (ELISA) to measure Spike-specific IgG antibodies

Serum IgG antibodies against SARS-CoV-2 Spike protein were measured by an ELISA developed and standardized in our laboratory [19]. Briefly, 96-well microplates (Immulon 4HBX, Thermo Scientific) were coated with recombinant NCP-CoV (2019-nCoV) Spike protein (S1 + S2 ECD) (Sino Biological #40589-V08B1) at 2 µg/mL for 1 h at room temperature. Plates were then washed with Tween-20 0.05% in phosphate-buffered saline (PBS) (PBST) and blocked with assay buffer (1% bovine serum albumin (BSA) in PBS) for 1 h at 37 °C. After blocking, all subsequent steps were performed by a DYNEX DS2® Automated ELISA system (DYNEX Technologies, Chantilly, VA, USA). First, serum samples diluted at 1:50,000 in assay buffer were added in duplicate and plates were incubated for 2 h. Next, plates were washed with PBST and 100 µL per well of a peroxidase-conjugated goat anti-human IgG (Jackson ImmunoResearch #109-036-098), diluted 1:10,000 in assay buffer, were added. After 1 h incubation, plates were washed, and a stabilized 3,3′,5,5′-tetramethylbenzidine (TMB) substrate (Sigma) was added to the wells. The enzymatic reaction was stopped after 20 min with a stop solution (1 M sulfuric acid), and absorbance at 450 nm was read by the DYNEX DS2 instrument.

Neutralizing antibodies were measured by the cPass neutralization ELISA assay, a surrogate plaque reducing neutralization test using the DYNEX Agility® Automated ELISA system. The kit relies on the disruption of the protein–protein interaction between receptor-binding domain-horseradish peroxidase (RBD-HRP) and human angiotensin-converting enzyme 2 (hACE2) by neutralizing antibodies against SARS-CoV-2. First, the samples are preincubated with the RBD-HRP to allow interaction and binding of the antibodies. The sample is then added to the precoated hACE2 protein-coated plate. The plate is then washed, and then TMB is added, producing the blue color. The stop solution is then added, and the wells read at 450 nm. A calibration curve is generated using the cPass standard using the established concentration, and sample results are calculated from the interpolated standard curve.

### ELISA to measure autoimmune antibodies

To measure MDA-specific IgG antibodies, ELISA plates were coated with MDA-modified BSA protein (MDA-BSA, MyBioSource MBS390120), at the concentration of 10 µg/mL. Control ELISA plates were coated with BSA at a concentration of 10 µg/mL. MDA-specific optical density (OD) values were calculated by subtracting BSA OD values from MDA-BSA OD values [21, 24].

To measure AD-specific IgG antibodies, we isolated the adipocytes from the subcutaneous AT of patients undergoing weight reduction surgeries (bilateral breast reduction), as previously described [23]. After isolation, the adipocytes were centrifuged in a 5415C Eppendorf microfuge (2000 rpm, 5 min). Total cell lysates were obtained using the M-PER (Mammalian Protein Extraction Reagent, ThermoFisher), according to the manufacturer’s instructions. Aliquots of the protein extracts were stored at −80 °C. Protein content was determined by Bradford [25]. AD at a concentration of 10 µg/mL were used to coat ELISA plates [21, 24].

For all tests, serum samples were diluted 1:1000 with sample buffer.

### ELISA to measure serum C-reactive protein (CRP)

Serum levels of CRP were measured using the commercially available kit R&D # DCRP00.

### Statistical analyses

We used one-way analysis of variance to examine differences between the four groups of participants (uninfected lean and obese, COVID lean and obese). Group-wise differences were analyzed afterward with Bonferroni’s multiple-comparisons test, with *p* < 0.05 set as a criterion for significance. *F* and *p* values generated after mixed-effects analyses were calculated and are reported in each figure legend. We used Student’s *t* tests (two-tailed) to examine differences between two groups. We used bivariate Pearson’s correlation analyses to examine relationships between variables. GraphPad Prism version 8.4.3 software was used to construct all graphs.

## Results

### Evaluation of Spike-specific IgG antibodies in serum samples of lean and obese COVID-19 patients, as compared to uninfected controls

**Fig. 1:**
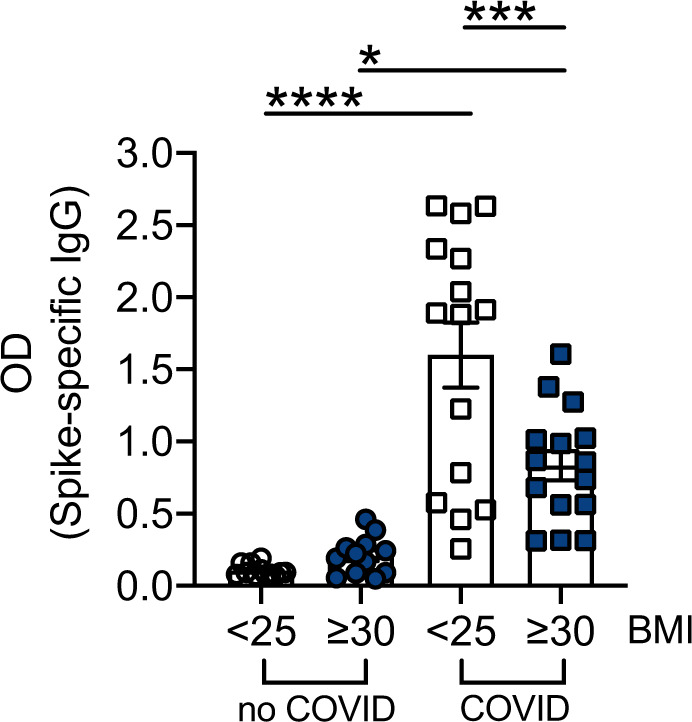
Evaluation of Spike-specific IgG antibodies in serum samples of lean and obese COVID-19 patients, as compared to uninfected controls. Bars show means ± SE (standard errors) of SARS-CoV-2 Spike-specific IgG antibodies (OD) measured by ELISA. Mean comparisons between groups were performed by one-way ANOVA (*F* = 23.76, *p* = 0.0029). *p < 0.05, ***p < 0.001, ****p < 0.0001.

The results for Spike-specific IgG antibodies in serum samples of 30 COVID-19 patients and 30 uninfected controls, age-, gender-, and BMI-matched, are shown in Fig. 1. Antibodies were measured by an ELISA previously developed and standardized in our laboratory [19]. Results show significantly lower levels of Spike-specific IgG antibodies in obese versus lean COVID-19 patients, confirming our published findings that Spike-specific IgG antibodies in serum are negatively associated with BMI in COVID-19 patients [19]. In addition, spike-specific IgG antibodies were detected at extremely low levels in serum samples isolated from uninfected lean and obese age-, gender-, and BMI-matched participants recruited before the pandemic.

### Evaluation of Spike-specific neutralizing IgG antibodies in serum samples of lean and obese COVID-19 patients

**Fig. 2:**
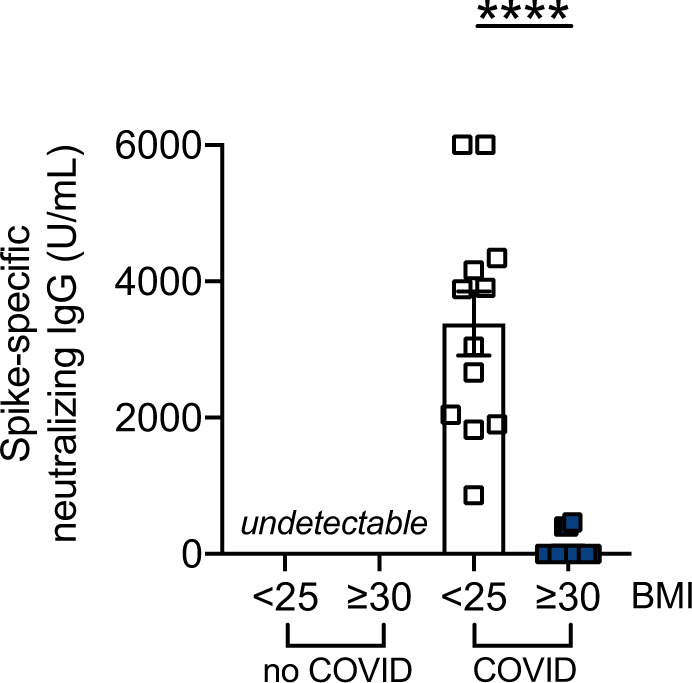
Evaluation of Spike-specific IgG neutralizing antibodies in serum samples of lean and obese COVID-19 patients. Neutralizing antibodies were measured by the cPass neutralization ELISA assay, a surrogate plaque reducing neutralization test (sVNT) using the DYNEX Agility® Automated ELISA system. This test allows the identification of neutralizing antibodies with high, medium, or low neutralization potential, corresponding to >5000, 1500–500, and <1500 U/mL, respectively. Bars show means ± SE (standard errors) of Spike-specific neutralizing IgG antibodies (U/mL). Mean comparisons between groups were performed by unpaired Student’s *t* test. ****p < 0.0001.

Results in Fig. 2 show that neutralizing antibodies were present in serum samples of all lean COVID-19 patients, whereas only a few obese COVID-19 patients had neutralizing antibodies.

### Evaluation of autoimmune IgG antibodies in serum samples of lean and obese COVID-19 patients, as compared to uninfected controls

**Fig. 3:**
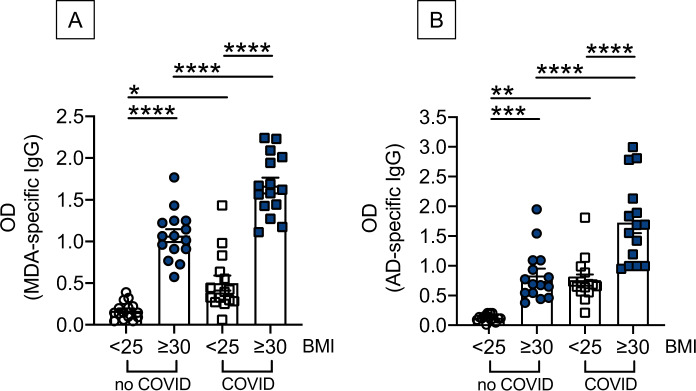
Evaluation of autoimmune IgG antibodies in serum samples of lean and obese COVID-19 patients, as compared to uninfected controls. Bars show means ± SE (standard errors) of anti-MDA (**A**) and anti-AD (**B**) IgG antibodies measured by ELISA. Mean comparisons between groups were performed by one-way ANOVA. **A** (*F* = 82.90, *p* < 0.0001). **B** (*F* = 32.41, *p* = 0.049). *p < 0.05, **p < 0.01, ***p < 0.001, ****p < 0.0001.

We evaluated the presence of IgG antibodies with two autoimmune specificities in serum samples of lean and obese COVID-19 patients as compared to uninfected controls. Results in Fig. 3 show that SARS-CoV-2 infection significantly increases serum levels of anti-MDA IgG antibodies (Fig. 3A) and anti-AD IgG antibodies (Fig. 3B) in both lean and obese patients, with a greater increase in lean versus obese patients. Fold increases in lean versus obese were 3.1 versus 1.6 for anti-MDA IgG antibodies and 6.4 versus 1.5 for anti-AD IgG antibodies. However, serum levels of all these antibodies were always higher in obese versus lean COVID-19 patients and uninfected controls.

### Association of autoimmune IgG antibodies with CRP

**Fig. 4:**
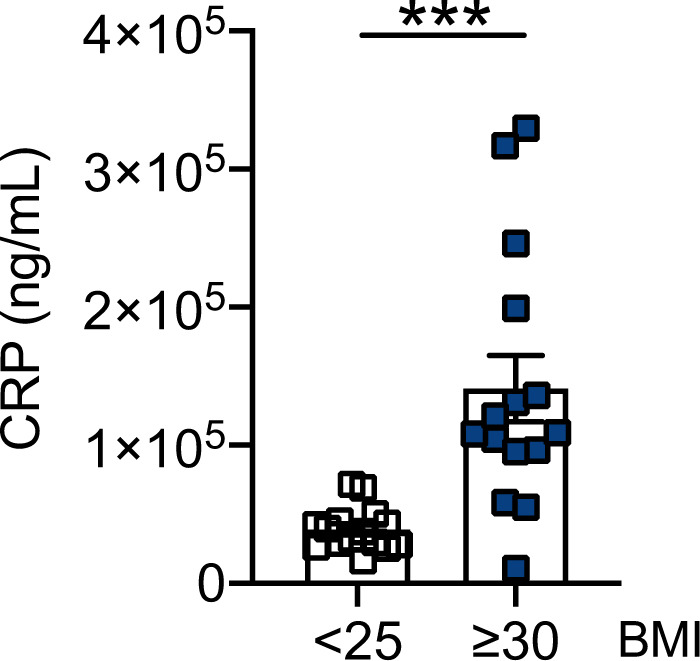
Serum levels of CRP are higher in obese versus lean COVID-19 patients. Bars show means ± SE (standard errors) of CRP serum levels measured by ELISA. Mean comparisons between groups were performed by unpaired Student’s *t* test. ***p < 0.001.

**Fig. 5:**
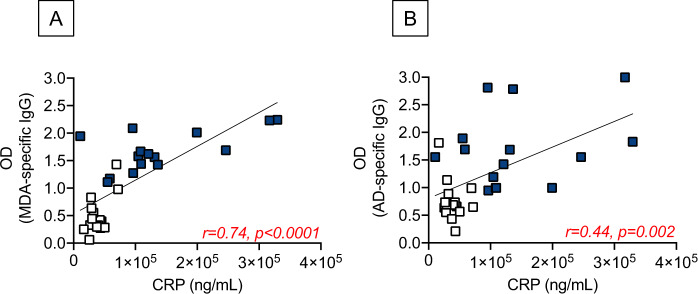
Serum levels of CRP are positively correlated with autoimmune IgG antibodies in lean and obese COVID-19 patients. Correlations of serum CRP with anti-MDA (**A**) and anti-AD (**B**) are shown. Symbol designations as in Fig. 3 (white squares: lean, blue squares: obese). Pearson’s regression coefficients and *p* values are indicated at the bottom of each figure.

We measured the association of anti-MDA and anti-AD with serum CRP, a marker of inflammaging previously found to be positively associated with autoimmune antibodies [20]. Results in Fig. 4 show, as expected, higher serum levels of CRP in obese versus lean COVID-19 patients. Furthermore, results in Fig. 5 show a positive association between CRP and autoimmune antibodies in our cohort of lean and obese COVID-19 patients.

## Discussion

The results from this study show that the majority of COVID-19 patients with obesity make almost undiscernible amounts of neutralizing anti-SARS-CoV-2 antibodies, suggesting that obese individuals may be at a higher risk to respond poorly to SARS-CoV-2 infection. Our results are in disagreement with another study in which obesity was associated with increased fever and multiple severe symptoms of disease caused by SARS-CoV-2 infection but not with dysfunctional B and T cell immune responses [18]. These differences may depend on the characteristics of the different cohorts recruited in the two studies. All of our recruited participants with obesity, however, made autoimmune antibodies. We measured two different autoimmune specificities, MDA and AD, because obesity has already been shown to be associated with increased oxidative stress and lipid peroxidation (measured by MDA) [26, 27] and increased fat mass (measured by adipocyte-derived proteins released in the AT, AD) [23].

The onset of autoimmunity has been associated with viral infections, and it has been suggested that SARS-CoV-2 could be a triggering factor for the development of a rapid autoimmune, autoinflammatory disease in genetically predisposed individuals as those with high systemic interleukin-6 [28, 29], similar to what has been found in SARS‐CoV, influenza, and dengue infections. In SARS‐CoV patients, high levels of serum autoantibodies specific for type-2 pneumocytes have been found, and these antibodies were highly cytotoxic [30]. In influenza patients, virus-induced autoantibodies against the alveolar and glomerular basement membrane induced the autoimmune disease called Goodpasture’s syndrome [31, 32]. In dengue patients, virus-induced autoantibodies specific for endothelial cells, platelets, and coagulatory molecules lead to abnormal activation or dysfunction [33]. In all these cases, molecular mimicry between viral and host proteins may explain the cross-reactivity of induced autoantibodies.

In the current pandemic, it has been shown that serum samples of adult COVID-19 patients contain antibodies that target the tissues of infected patients instead of targeting the disease-causing virus. Anti-phospholipid, anti-type-I interferons, anti-nuclear antibodies, and rheumatoid factor have been found in a large number of COVID-19 patients and linked to severe disease as they may inactivate critical components of the antiviral response [20]. These findings have suggested that SARS-CoV-2 infection, following dissemination of the virus through the blood, may induce severe tissue damage, cell death, and release of intracellular self-antigens not previously known as disease-causing autoantigens, leading to a self-tolerance breakdown. We cannot exclude the possibility that the virus may also trigger autoimmunity through other mechanisms such as molecular mimicry between viral proteins and self-antigens or reactivation of local viral or bacterial pathogens that have not been removed due to dysfunctional immune responses in infected patients. We expected the secretion of autoimmune IgG to be increased in obese patients. However, our results have clearly shown that SARS-CoV-2 infection significantly increases serum levels of anti-MDA and anti-AD IgG antibodies more in lean than in obese patients. Serum levels of these antibodies, however, were always higher in obese versus lean COVID-19 patients. These results suggest that the infection has induced de novo autoimmune responses and self-tolerance breakdown following severe tissue damage in lean patients and demonstrates a heightened response in obese patients in which these processes are already occurring.

Early reports have shown that autoimmune IgG antibodies in adult COVID-19 patients are correlated with serum levels of CRP [20]. CRP is a marker of pathogen-driven pulmonary inflammation, embolism, and disseminated intravascular coagulation, all characteristics of COVID-19, and therefore CRP is a predictive marker of adverse health outcomes [34–37]. Subsequent results from the same group have shown a positive correlation between disease severity and self-tolerance breakdown, with patients with the highest serum levels of CRP also having increased amounts of autoimmune antibodies and intensities of autoreactive tests [38]. Also, CRP has been shown to correlate with blood frequencies of the subset of extrafollicular B cells that is expanded in critically ill COVID-19 patients as well as in lupus autoimmune patients [39]. These results have suggested that CRP is an effective predictor not only of disease severity but also of pathogenic autoimmune antibodies in COVID-10 patients.

We also measured serum Spike-specific IgG antibodies in our cohorts of lean and obese COVID-19 patients and uninfected controls. These antibodies were found to be significantly lower in obese as compared to lean COVID-19 patients, confirming our previously published findings that BMI is negatively associated with anti-Spike IgG serum levels [19] and consistent with the knowledge that obesity is associated with inflammaging [2] and metaflammation [40] both of which are negatively associated with a functional immune system [41]. In addition, our previously published work has shown that overweight/obesity decrease the serum antibody response to the influenza vaccine in young and elderly individuals [12]. As expected, serum Spike-specific IgG antibodies were not found in lean and obese age-, gender-, and BMI-matched controls.

In conclusion, our results highlight the importance of identifying protective (neutralizing) versus pathogenic (autoimmune) antibodies in COVID-19 patients with obesity. In addition, similar autoimmune antibodies may also be secreted following COVID-19 vaccination. However, the reactogenicity of lipid nanoparticle-formulated COVID-19 mRNA vaccines in individuals with obesity, characterized by dysregulation of immune responses, has not been investigated yet. Therefore, evaluating the quality of the antibody response of obese COVID-19 patients is crucial to protect this vulnerable population at higher risk of responding poorly to infection with SARS-CoV-2 and vaccination against SARS-CoV-2 lean controls. Although our study has the limitation of having a small number of recruited participants, we believe our results have a high impact showing that obese individuals may be at a higher risk to respond poorly to SARS-CoV-2 infection.
